# Realization of mid-infrared graphene hyperbolic metamaterials

**DOI:** 10.1038/ncomms10568

**Published:** 2016-02-04

**Authors:** You-Chia Chang, Che-Hung Liu, Chang-Hua Liu, Siyuan Zhang, Seth R. Marder, Evgenii E. Narimanov, Zhaohui Zhong, Theodore B. Norris

**Affiliations:** 1 Center for Photonics and Multiscale Nanomaterials, University of Michigan, 2200 Bonisteel Blvd., Ann Arbor, Michigan 48109, USA; 2 Department of Physics, University of Michigan, 450 Church St, Ann Arbor, Michigan 48109, USA; 3 Department of Electrical Engineering and Computer Science, University of Michigan, 1301 Beal Avenue, Ann Arbor, Michigan 48109, USA; 4 School of Chemistry and Biochemistry, Georgia Institute of Technology, 901 Atlantic Drive, Atlanta, Geogia 30332, USA; 5 School of Electrical and Computer Engineering and Birck Nanotechnology Center, Purdue University, 1205 West State Street, West Lafayette, Indiana 47907, USA

## Abstract

While metal is the most common conducting constituent element in the fabrication of metamaterials, graphene provides another useful building block, that is, a truly two-dimensional conducting sheet whose conductivity can be controlled by doping. Here we report the experimental realization of a multilayer structure of alternating graphene and Al_2_O_3_ layers, a structure similar to the metal-dielectric multilayers commonly used in creating visible wavelength hyperbolic metamaterials. Chemical vapour deposited graphene rather than exfoliated or epitaxial graphene is used, because layer transfer methods are easily applied in fabrication. We employ a method of doping to increase the layer conductivity, and our analysis shows that the doped chemical vapour deposited graphene has good optical properties in the mid-infrared range. We therefore design the metamaterial for mid-infrared operation; our characterization with an infrared ellipsometer demonstrates that the metamaterial experiences an optical topological transition from elliptic to hyperbolic dispersion at a wavelength of 4.5 μm.

## Abstract (web summary)

The use of graphene can provide another useful building block for metamaterials. Here, Chang *et al*. have explored the experimental realization of a mid-infrared hyperbolic metamaterial, in which the role of the metal in providing a conducting layer is taken over by graphene.

## Graphical abstract

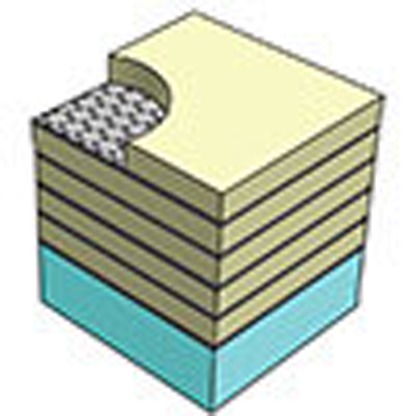

Hyperbolic metamaterials (HMMs) are artificially structured materials designed to attain an extremely anisotropic optical response, in which the permittivities associated with different polarization directions exhibit opposite signs^1^,^2^,^3^. Such anisotropic behaviour results in an isofrequency surface in the shape of a hyperboloid, which supports propagating high *k*-modes and exhibits an enhanced photonic density of states. Many interesting applications have been enabled by HMMs. For example, the spontaneous emission rate of quantum emitters can be modified if they are brought close to a HMM^4^, and similarly, the scattering cross-section of small scatterers near a HMM is enhanced^5^. The near-field radiative heat transfer associated with HMMs becomes super-Planckian^6^. Also, the propagating high *k*-modes supported by HMM are exploited to achieve sub-diffraction-limited images using a hyperlens^7^. Some natural materials such as bismuth, graphite and hexagonal boron nitride exhibit hyperbolic dispersion in specific spectral ranges^8^,^9^,^10^, while artificial HMMs are most commonly realized with two categories of structures such as metal-dielectric multilayers^4^,^7^ and metallic nanorod arrays^11^. The former structure can be fabricated layer by layer using vapour deposition, and the latter is often obtained by electrochemical deposition of a metal on porous anodic aluminium oxide. In both cases, metal is the essential element to provide the conducting electrons that make the extreme anisotropicity possible. Metals can also be replaced by doped semiconductors for realizing HMMs in the infrared range^12^.

In this paper, we explore the realization of a particular HMM, in which the role of the metal in providing a conducting layer is taken over by graphene^13^,^14^,^15^,^16^,^17^,^18^,^19^,^20^,^21^. Graphene is a two-dimensional (2D) semi-metal with a thickness of only one atom^22^,^23^. It has been shown that doped graphene is a good infrared plasmonic material in terms of material loss^24^. As a truly 2D material that only conducts in the plane, graphene by nature has the anisotropicity required for HMMs. As the thinnest material imaginable, graphene also makes an ideal building block for multilayer structures, as it enables the minimum possible period and therefore the highest possible cutoff for the high *k*-modes^14^,^25^, which has been limited in metal and semiconductor-based HMMs by the non-negligible thickness of those materials. The conductivity of graphene, unlike that of metals, can be effectively modulated by electrical gating (see Supplementary Fig. 1 and Supplementary Note 1) or optical pumping^26^,^27^. This unique advantage has been demonstrated in other graphene-based metamaterials^28^, and can potentially be exploited to realize a tunable HMM, in which the photonic density of states can be controlled electronically on demand. In addition, graphene shows much richer optoelectronic behaviour than metals, and the massless Dirac quasi-particles in graphene also give rise to very different carrier dynamics compared with other semiconductors. Various photodetection mechanisms, such as thermoelectric, bolometric, photovoltaic, photo-gating and photo-Dember effects, have been demonstrated with graphene^29^,^30^,^31^,^32^. Graphene multilayer structures can therefore serve as a unique platform in optoelectronics, incorporating the unusual photonic behaviour of HMMs into graphene detectors or other optoelectronic devices. For example, an ultrathin super-absorber enabled by HMM could be incorporated into graphene detectors to enhance the light absorption^18^. A brief summary of this report is as follows. The design criterions and material choices for realizing the graphene HMM are discussed. Chemical vapour deposited (CVD) graphene is identified as a good practical choice in the mid-infrared range when it is heavily doped. A chemical doping method is developed to obtain the desired high carrier density and ellipsometry is used to characterize the optical conductivity of monolayer graphene. The metamaterial with multilayer structure is fabricated by repetitive graphene transfer and dielectric deposition. We characterize the effective permittivities of the fabricated metamaterial with ellipsometry to demonstrate the hyperbolic dispersion in the mid-infrared range.

## Results

### Design of graphene HMM

**Figure 1:**
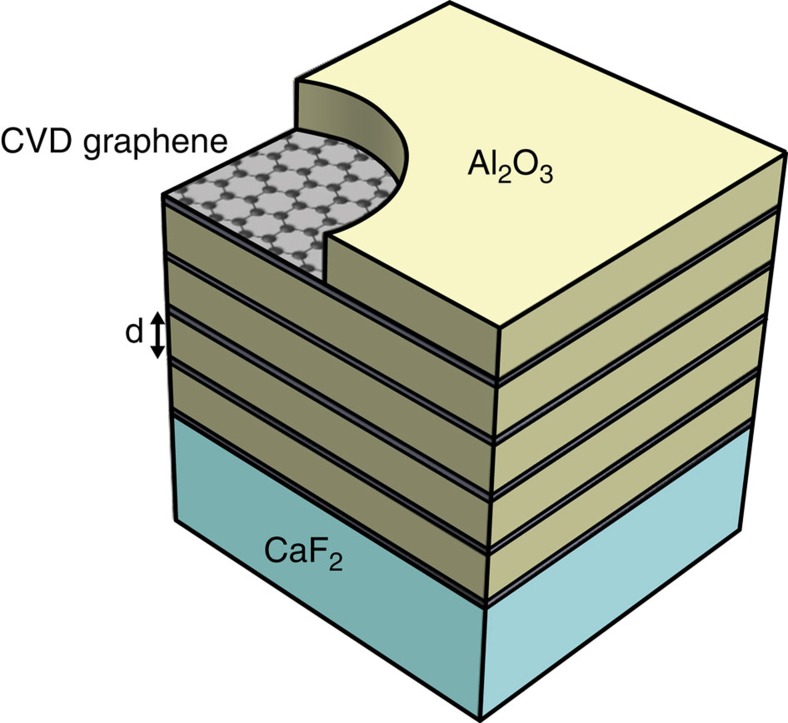
The schematic representation of the graphene-dielectric multilayer structure that turns into a HMM at mid-infrared frequencies. It consists of five periods of alternating CVD graphene sheets and Al_2_O_3_ layers on a CaF_2_ substrate. The thickness *d* of the Al_2_O_3_ layer is ∼10 nm.

Figure 1 shows the structure of the graphene-based HMM, which consists of alternating dielectric and graphene layers. Similar graphene-dielectric multilayer structures have been proposed and analysed theoretically by different groups and shown to function as a HMM operating at terahertz (THz) and mid-infrared frequencies^13^,^14^,^15^,^16^,^17^,^18^,^19^,^20^,^21^. Various applications have also been discussed. For example, in our previous work we have calculated theoretically the Purcell factor of a graphene-based HMM with a finite number of layers^17^, and we have simulated numerically the light coupling from free space into a graphene-based HMM slab with a metallic grating^18^. In spite of the large body of theoretical work on graphene-based HMM, no experimental demonstrations have yet been reported, the primary reason being the challenge in obtaining a sufficiently high level of doping in the graphene layers in the required multilayer structure.

The graphene-dielectric multilayer structure can be homogenized and viewed as a metamaterial using the effective medium approximation (EMA). The effective out-of-plane and in-plane permittivities of this metamaterial can be derived by taking the long-wavelength limit of the Bloch theory^13^,^14^,^15^,^16^:


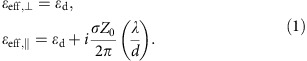


Here *ɛ*_d_ is the permittivity of the dielectric layer, *d* is the dielectric thickness and *σ* is the optical conductivity of graphene. *Z*_0_ is the vacuum impedance. Here graphene, as a 2D material, is treated as an infinitely thin layer described by its in-plane sheet conductivity. As indicated by equation 1, the graphene-dielectric multilayer system forms a uniaxial anisotropic metamaterial. *ɛ*_eff,⊥_ is the same as the constituent dielectric and is always positive. On the other hand, the real part of *ɛ*_eff,||_ becomes negative if


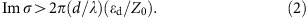


When this criterion is satisfied, the isofrequency surface becomes a hyperboloid and we obtain HMM. Such an isofrequency surface allows the existence of propagating high *k*-modes, which can be traced back to the coupled plasmon modes in the graphene-dielectric multilayer structure^17^. The criterion described by equation 2 determines the wavelength at which the optical topological transition between elliptical and hyperbolic dispersions occurs^4^.

**Figure 2:**
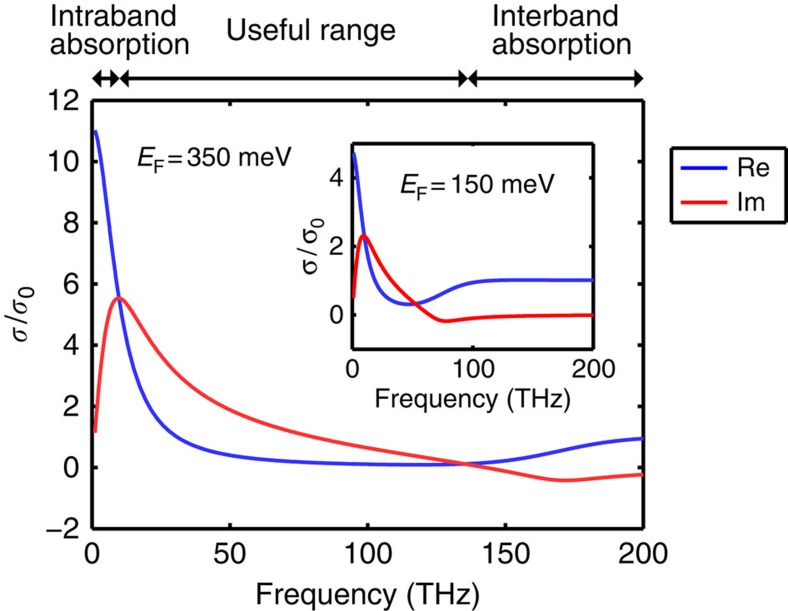
The theoretical optical conductivity of graphene. It is plotted with *E*_F_=350 meV and *ℏγ*=40 meV. These numbers correspond to heavily doped CVD graphene. At the high-frequency end of the spectrum, graphene is lossy because of the interband absorption. At the low-frequency end, graphene is again lossy because of the intraband free-carrier absorption. There is a useful spectral range in between, where the imaginary part of the optical conductivity exceeds the real part. In this particular example, the useful wavelengths range from 2 to 30 μm in the mid-infrared range. The inset shows another example of lightly doped CVD graphene with *E*_F_=150 meV and *ℏγ*=40 meV. The useful wavelength range is smaller when the doping is lower.

While most previous theoretical work has concentrated on using high-mobility graphene that may be obtained from mechanically exfoliated or epitaxially grown samples, we use CVD graphene because it is the most realistic choice for practical fabrication of a multilayer structure^33^. Growth of large-area CVD graphene is well established, and it can be transferred onto arbitrary surfaces using poly(methyl methacrylate) (PMMA) as the carrier material. In spite of its advantage in fabrication, CVD graphene often has a higher degree of disorder, which is typically manifested by a reduced mobility (usually on the order of thousands cm^2^ V^−1^ s^−1^). As a result of the lower crystal quality, the stronger carrier scattering in typical polycrystalline CVD graphene enhances the free-carrier absorption at THz frequencies, which can be understood from the theoretical optical conductivity of graphene^34^,^35^,^36^


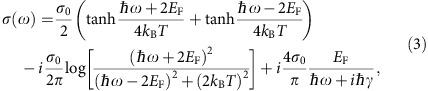


where *σ*_0_ equals to *e*^2^/(4*ℏ*), *E*_F_ is the Fermi energy relative to the Dirac point and *γ* is the intraband scattering rate. In this expression, the first two terms correspond to interband transitions, while the third term is the Drude-like intraband conductivity. Figure 2 shows a plot of the theoretical optical conductivity given by equation 3 with parameters typical for doped polycrystalline CVD graphene. To realize a good HMM, we need graphene with a large positive imaginary conductivity to interact with light, but with a small real conductivity to minimize the material loss. As indicated by Fig. 2, graphene is lossy at high frequencies when *ℏω*>2*E*_F_ because of interband transitions. On the other hand, at low frequencies when *ℏω*≲*ℏγ*, graphene also exhibits a large loss because of the intraband free-carrier absorption enabled by scattering. Because CVD graphene typically has a *ℏγ* of tens of meV, it is a lossy material at THz frequencies^37^. As shown by Fig. 2, however, there is a spectral range between the two lossy regions, such that the imaginary part of the conductivity exceeds the real part. As this spectral range lies in the mid-infrared part of the spectrum, CVD graphene-based HMM operates better in the mid-infrared than the THz region. Also, Fig. 2 indicates that doping can improve the properties of graphene for realizing a HMM. A large *E*_F_ can turn off the interband absorption by the Pauli blocking and increase the Im*σ* required for achieving negative *ɛ*_eff,||_. Furthermore, doping can also suppress the intraband scattering by screening charged impurities^37^,^38^.

### Characterization of the optical conductivity of grapheme

Because graphene is the key building block of the metamaterial, it is important to have an accurate measurement on the optical conductivity of the actual CVD graphene layers used to fabricate the sample. Although the theoretical optical conductivity given by equation 3 provides a good guideline for designing the graphene HMM, real CVD graphene layers can have imperfections or extrinsic properties that are not taken into account by equation 3. We therefore need to characterize actual graphene samples and examine the scope of validity of equation 3.

**Figure 3:**
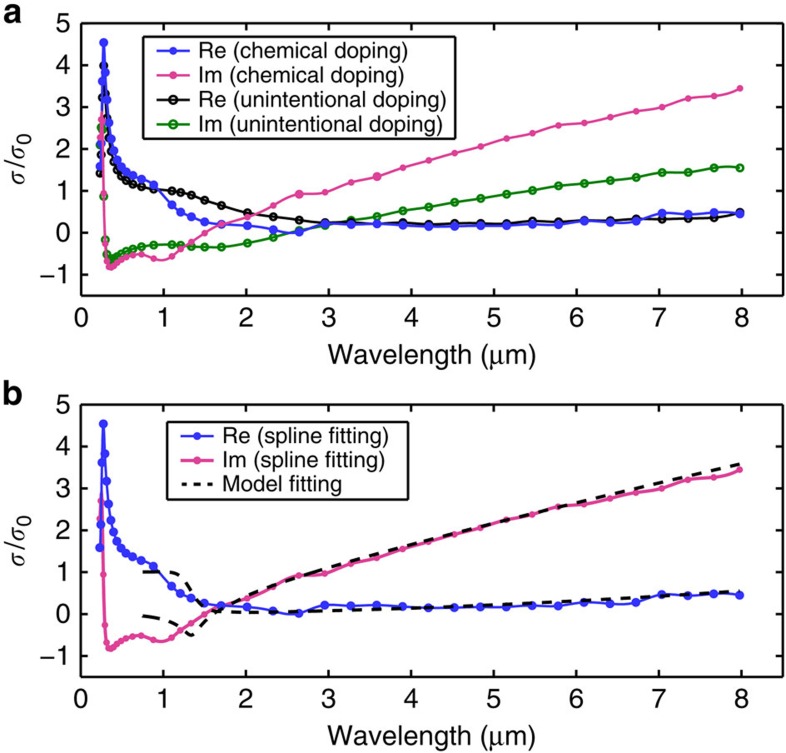
The optical conductivity of CVD graphene measured by ellipsometry. (**a**) The real and imaginary part of the optical conductivity of the chemically doped CVD graphene (blue and magenta curves) and the unintentionally doped CVD graphene (black and green curves). These curves are mathematically expressed by cubic splines, and the markers denote the control points of the splines. The chemically doped CVD graphene has a larger imaginary conductivity in the mid-infrared range. (**b**) The real and imaginary part of the optical conductivity of the chemically doped CVD graphene. The blue and magenta curves are obtained by fitting with cubic splines, and the black dash lines are obtained by using the model given by equation 3. The model fitting is consistent with the spline fitting in the mid-infrared range. The extracted *E*_*F*_ and *ℏγ* from the model fitting are 460 and 23 meV, respectively, which corresponds to a mobility of ∼2,000 cm^2^ V^−1^ s^−1^.

In our previous work, we have developed a technique based on ellipsometry to measure the optical conductivity of truly 2D materials^39^. In this technique, the analysis used in conventional ellipsometry is modified to handle the infinitely thin 2D material whose properties are fully described by the 2D optical conductivity. To characterize actual CVD graphene samples with this technique, we have prepared two kinds of samples, unintentionally doped and the chemically doped CVD graphene, on CaF_2_ substrates by the standard PMMA transfer method. Even without chemical treatment, unintentionally doped CVD graphene is p-type because of adsorbed gas molecules and residual ammonium persulfate from the transfer process^40^,^41^. The chemically doped CVD graphene is prepared by a solution process that leaves a sub-monolayer of Tris (4-bromophenyl)ammoniumyl hexachloroantimonate (also known as ‘magic blue'), a somewhat air-stable p-type dopant, on the surface (see Methods section, Supplementary Fig. 2 and Supplementary Note 2)^42^,^43^. Figure 3a shows the optical conductivities of both samples measured with ellipsometry. The optical conductivities shown here are mathematically described by cubic splines without assuming an *a priori* theoretical expression like equation 3. Consistent with Fig. 2, in the mid-infrared range the chemically doped graphene has a larger imaginary conductivity, which is necessary for creating the extreme anisotropicity in the metamaterial.

Although the spline-fitted conductivity of actual CVD graphene sample shown in Fig. 3a is useful in many applications, a conductivity model based on a theoretical expression such as equation 3 provides more physical insight and requires fewer unknown parameters to perform the fit. The latter is important when we want to parameterize the homogenized metamaterial, which will be discussed in next section. In Fig. 3b, we examine how well equation 3 works for our chemically doped CVD graphene samples. In fitting the ellipsometer data, we express the optical conductivity *σ*(*ω*) by the model given by equation 3 with *E*_F_ and γ being the only two unknown fitting parameters. We also show in the same figure the spline-fitted conductivity obtained from the same set of data. It is apparent that the resulting conductivity based on equation 3 overlaps very well with the spline-fitted conductivity throughout the mid-infrared range, assuring the validity of using equation 3 for the mid-infrared metamaterial. We extract from the fit that *E*_F_=460 meV and *ℏγ*=23 meV. A mobility of ∼2,000 cm^2^ V^−1^ s^−1^ can be calculated from these numbers using the relationship 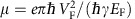, where *μ* is the mobility and *V*_F_ is the Fermi velocity.

In the mid-infrared range, the optical conductivity is mostly determined by intraband transitions, which are described by the Drude-like term in equation 3. Our result is consistent with ref. 37, which shows that the Drude model can successfully fit the measured absorption spectrum of CVD graphene over a broad range of infrared wavelengths. We do not apply equation 3 in the ultraviolet to visible wavelength range because the many-body correction has been shown to be important^44^,^45^. There is some discrepancy between the model and spline fits in the near-infrared (∼1.5 μm, that is, near the wavelength corresponding to interband transitions close to the Fermi level). The origin of this discrepancy is not quantitatively understood, but may be related to spatial inhomogeneity in the Fermi energy or other disorder effects. Since the optical topological transition wavelength of our HMM is very far from this spectral region, and the fit is excellent over the entire mid-infrared range, the failure of the simple model in the near-infrared region does not affect the behaviour of the material in the mid-infrared, which is the region of concern in this work. Equation 3 thus provides an excellent description for the mid-infrared conductivity. Other imperfections that are typically present in transferred CVD graphene samples, such as the existence of small multilayer graphene patches and holes (see Supplementary Fig. 3 and Supplementary Note 3), can also contribute to the deviations observed in Fig. 3b (ref. 46).

### Measurement of the effective permittivity of graphene HMM

We have fabricated the multilayer structure shown in Fig. 1, which consists five periods of alternating CVD graphene and Al_2_O_3_. The CVD graphene is transferred by the PMMA method and doped with Tris (4-romophenyl) ammoniumyl hexachloroantimonate (‘magic blue'). The Al_2_O_3_ dielectric layer is grown by atomic layer deposition (ALD). We choose Al_2_O_3_ as the dielectric material, because it has negligible loss at the mid-infrared wavelengths up to 8 μm. The dielectric thickness is chosen to be ∼10 nm to create an optical topological transition in the mid-infrared range.

**Figure 4:**
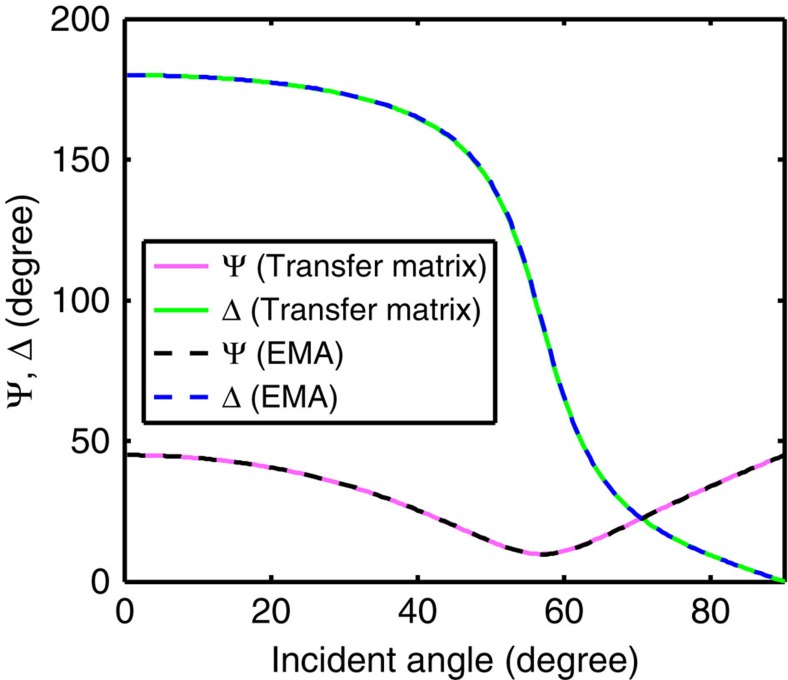
Calculation of ellipsometric angles with exact transfer-matrix method and EMA. Ellipsometric angles Ψ and Δ are defined by *r*_p_/*r*_s_=(tanΨ)*e*^*i*Δ^, where *r*_p_ and *r*_s_ are the reflection coefficients for p and s light, respectively. They are the quantities an ellipsometer measures. The transfer-matrix method calculates the response of five periods of graphene-dielectric multilayer structure, while the EMA simulates a homogenized anisotropic layer with the permittivities given by equation 1. This calculation shows that the EMA is an accurate approximation for the structure. The wavelength used in this simulation is 6 μm. The material properties are *ɛ*_d_=2.1 and *σ*=(0.43+1.98*i*) *σ*_0_. Thickness *d*=10 nm. The substrate has a refractive index of 1.39.

To characterize the metamaterial, we use infrared ellipsometry, which is appropriate to probe the effective permittivity of a metamaterial, since it measures the sample with free-space plane waves and the transverse wave vector (*k*_0_sin*θ*) associated with the free-space plane waves is very small (*k*_0_sin*θd*<<1 , where *θ* is the angle of incidence). We are therefore probing the low *k*-modes of the metamaterial, ensuring the validity of the long-wavelength approximation. Although the long-wavelength approximation is evidently satisfied for our metamaterial (*d*/*λ*<1/300 in our case), we still need to confirm the validity of the EMA with a rigorous transfer-matrix calculation, since the EMA is derived for an infinite periodic system, while our metamaterial has only five periods. In Fig. 4 we show the transfer-matrix calculation of five periods of graphene-dielectric multilayer structure and the EMA calculation with the structure homogenized into an anisotropic layer, with the permittivities of the homogenized anisotropic layer given by equation 1. Here we calculate the ellipsometric angles Ψ and Δ, the quantities an ellipsometer acquires directly, at different incident angles. Ψ and Δ are defined by *r*_p_/*r*_s_=(tanΨ)*e*^*i*Δ^, where *r*_p_ and *r*_s_ are the reflection coefficients for p and s light, respectively. Numbers used in the simulation are chosen according to measured material properties of the individual layers. As demonstrated by Fig. 4, the two methods give very close results, confirming that the five-period graphene-dielectric structure, in the low *k*-regime probed by ellipsometry, can be accurately treated as a metamaterial with the effective permittivities given by equation 1. In fact, in the low *k*-regime, even one period of the graphene-dielectric unit cell can be homogenized by the same EMA formula given by equation 1 and still reproduce the optical properties accurately (see Supplementary Figs 4 and 5 and Supplementary Note 4). However, the high *k*-regime is where the real interest of HMM lies, and as discussed in Supplementary Note 4, the high *k* optical properties depend on the number of unit cells in the metamaterial. The five-period structure in our experimental realization of graphene HMM is chosen to create desirable high *k* optical properties.

**Figure 5:**
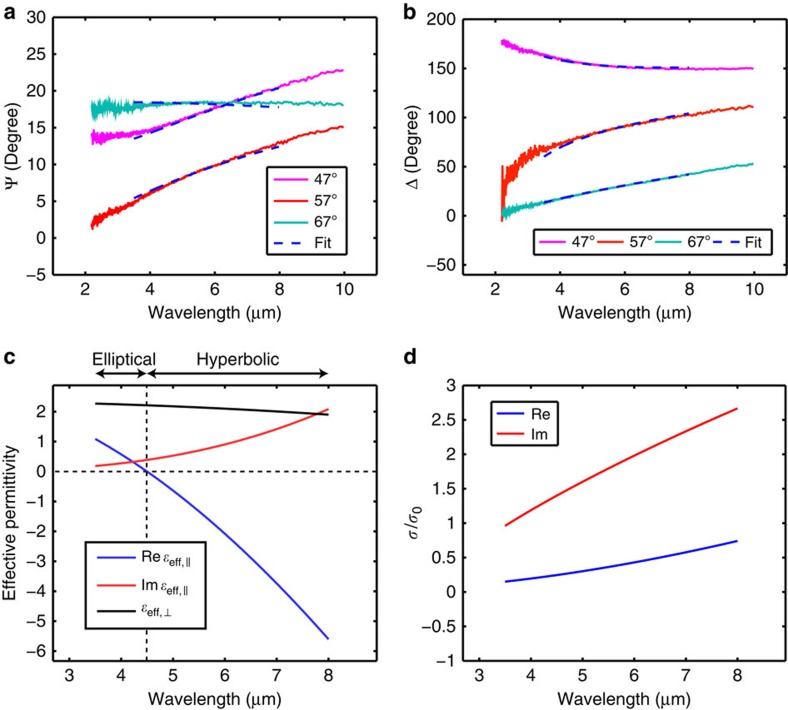
Extraction of the effective permittivity of the graphene HMM. (**a**,**b**) The ellipsometric angles Ψ and Δ acquired from the graphene-dielectric multilayer structure. The measurement is performed at incident angles of 47°, 57° and 67°. The blue dash lines show the fitting by homogenizing the multilayer structure into a metamaterial with the effective permittivities given by equation 1. We extract from the fitting that *E*_F_=365 meV and *ℏγ*=41 meV. (**c**) The extracted effective permittivity of the metamaterial, which exhibits an optical topological transition from elliptical to hyperbolic dispersion at 4.5 μm. When the wavelength is at 6 μm, *ɛ*_eff,||_ equals 2.1+0.9*i* and *ɛ*_eff,⊥_ equals 2.1. (**d**) The extracted optical conductivity of the constituent CVD graphene in the metamaterial.

The results of infrared ellipsometry, ellipsometric angles Ψ and Δ for our HMM sample, are shown in Fig. 5a,b, from which we extract the effective permittivities by fitting the acquired data. A robust and physical fitting in ellipsometry requires correct prior knowledge about the sample parameters, which allows us to use a minimal number of unknowns. Since our simulation in Fig. 4 demonstrates that the EMA is an accurate description for the multilayer structure, we can apply equation 1 in fitting the data. More precisely, we fit the experimental data to a layer of an anisotropic material on a CaF_2_ substrate with the permittivities of the anisotropic material given by equation 1. In equation 1, we know everything except the optical conductivity of graphene *σ*, as we have measured the thickness *d* independently after depositing each Al_2_O_3_ layer, and we have measured the refractive index of the ALD-grown Al_2_O_3_ in the relevant spectral range independently on a reference sample (see Methods section). Furthermore, as shown by Fig. 3b, considering the mid-infrared range with only the intraband response, the expression of equation 3 is a good description for the optical conductivity of the actual CVD graphene layers. Therefore, we can apply equation 3 and parameterize the optical conductivity with only *E*_F_ and γ. As a result of this independent knowledge of the sample, only two unknowns, *E*_F_ and γ, are sufficient to fit the experimental data of the multilayer metamaterial.

The fitted results of the ellipsometric angles Ψ and Δ are plotted as the blue dash lines in Fig. 5a,b. We restrict the wavelengths range of the fitting to 3.5–8 μm, where the lower bound is limited by the requirement of intraband-only response in the application of equation 3, and the upper bound is because of the limited transparent spectral range of Al_2_O_3_. As shown by Fig. 5, we are able to reproduce all six Ψ and Δ curves acquired at different incident angles with only two free parameters in the fitting. The extracted *E*_F_ is 365 meV, and the extracted *ℏγ* is 41 meV. The extracted *E*_F_ is lower than the value we typically obtain from chemically doped monolayer CVD graphene, because some dopants are lost in the ALD process because of the vacuum environment and the elevated temperature. The obtained scattering rate *ℏγ* is higher than the value of graphene on CaF_2_ substrate shown in Fig. 3. This can be explained by the fact that the carrier scattering in graphene depends on the surrounding environment, from which we conclude that sandwiching graphene between Al_2_O_3_ increases the carrier scattering.

Figure 5c shows the effective permittivity of the graphene metamaterial given by the extracted values of *E*_F_ and γ. *ɛ*_eff,⊥_ is always positive because it equals the permittivity of Al_2_O_3_. On the other hand, the real part of *ɛ*_eff,||_ changes from positive to negative at 4.5 μm indicating an optical topological transition from an elliptical metamaterial to a HMM. This graphene metamaterial is therefore a transverse epsilon-near-zero metamaterial at the wavelength of 4.5 μm (ref. 14). The imaginary part of *ɛ*_eff,||_ is several times smaller than the real part in most of the spectral range with hyperbolic dispersion, indicating that the loss of this HMM is reasonably low. In Fig. 5d, we plot the optical conductivity of the constituent graphene sheet of the metamaterial using the extracted *E*_F_ and γ.

## Discussion

Our characterization by the infrared ellipsometry demonstrates that the graphene-dielectric multilayer structure indeed experiences an optical topological transition from an elliptical to a hyperbolic dispersion in the mid-infrared range, confirming the theoretical predictions in previous works^13^,^14^,^15^,^16^,^17^,^18^,^19^,^20^,^21^. Our metamaterial sample has an optical topological transition at a wavelength of 4.5 μm, and maintains good hyperbolic properties up to 8 μm. The upper bound of the wavelength range is limited by the absorption in Al_2_O_3_ and CVD graphene. While the absorption in the dielectric layer can be overcome by replacing Al_2_O_3_ with other infrared transparent materials such as ZnSe, the absorption in CVD graphene is limited by the quality of graphene. Recently, there have been reports of the growth of large-area CVD graphene with the quality of a single crystal^47^, and new transfer process for CVD graphene without degrading the mobility^48^. With higher quality CVD graphene, the intraband absorption resulted from scattering could potentially be suppressed. The transition wavelength, as determined by equation 2, can be shifted by choosing the dielectric thickness or controlling the doping of graphene. The latter is especially useful if it can be done by the electrical gating. Shifting the transition wavelength farther into the infrared can be done by using lightly doped graphene or thicker dielectric. We have also realized a graphene HMM with the same structure except that the CVD graphene layers were not chemically doped (see Supplementary Fig. 6 and Supplementary Note 5), resulting in a transition wavelength red-shifted to 7.2 μm. On the other hand, blue shifting the transition wavelength is limited by the highest doping and the thinnest dielectric layers achievable in practice. While the structure reported in this work has only five periods, the procedure developed here can be repeated to scale up the graphene HMM. Some applications of HMMs do not require a large number of periods; for example, only a few periods is sufficient to produce a Purcell factor close to a semi-infinite structure, according to the theoretical calculations in ref. 17.

## Methods

### Sample fabrication

The graphene-dielectric multilayer structure with five periods is fabricated on a CaF_2_ wedge. The CVD graphene is grown on copper foil (Graphenea Inc) and transferred to the substrate using the standard PMMA transfer technique^33^,^46^. The copper foil is etched using an ammonium persulfate solution. The size of the CVD graphene we transfer is ∼10 mm by 10 mm. After transferring each graphene layer, we dope the graphene by soaking the sample in a 0.25 mM solution of Tris (4-bromophenyl)ammoniumyl hexachloroantimonate ‘magic blue' in dichloromethane for 10 min, and then rinse the sample with dichloromethane (see Supplementary Fig. 2 and Supplementary Note 2). The Al_2_O_3_ dielectric layer is deposited by the ALD at 150 °C using trimethylaluminium as the Al precursor and H_2_O as the oxygen precursor. The number of cycles used in the ALD process is calibrated to grow ∼10 nm of Al_2_O_3_ on graphene, with the thickness characterized by an ellipsometer (Woollam M-2000). The procedure is repeated to fabricate five periods of the graphene-Al_2_O_3_ unit cell. We have also confirmed that the chemical doping with Tris (4-bromophenyl) ammoniumyl hexachloroantimonate does not affect the Al_2_O_3_ layer and the substrate. We have found that although nitric acid can also p-dope graphene effectively^37^,^39^, it is not a good dopant for making the multilayer structure because of damage to the thin Al_2_O_3_ layer. The substrate is wedged to avoid back side reflection in the ellipsometry measurement. We also characterize the graphene-dielectric multilayer structure with the Woollam M-2000 ellipsometer after depositing each Al_2_O_3_ layer and after transferring each graphene layer. With the acquired ellipsometry data, we extract an average Al_2_O_3_ thickness of 10.4 nm.

### Ellipsometry characterization

The optical conductivity of monolayer graphene is measured by the ellipsometric analysis method described in ref. 39. Two ellipsometers designed for different spectral ranges, Woollam M-2000 and Woollam IR-VASE, are used for the wavelengths from 230 nm to 1.64 μm and the wavelengths above 2 μm, respectively. The data are acquired at three angles of incidence: 47°, 57° and 67°. The spot sizes of M-2000 and IR-VASE are 3 mm by 5.5 mm and 8 mm by 20 mm, respectively when the incident angle is 57°. We mask the samples for the IR-VASE measurement because the spot size is larger than the graphene area. To obtain the refractive index of Al_2_O_3_, we have prepared a sample with ALD-grown Al_2_O_3_ film on a CaF_2_ wedge. We measure the sample with both ellipsometers, and fit the refractive index of Al_2_O_3_ with the Sellmeier equation. The measurement of the effective permittivities of the graphene-dielectric multilayer structure is performed by the IR-VASE ellipsometer with the same settings described above.

## Additional information

**How to cite this article:** Chang, Y.-C. *et al*. Realization of mid-infrared graphene hyperbolic metamaterials. *Nat. Commun.* 7:10568 doi: 10.1038/ncomms10568 (2016).

## Supplementary Material

Supplementary Information Supplementary Figures 1-6, Supplementary Notes 1-5 and Supplementary References.
