# Supplementary material for: Realization of mid-infrared graphene hyperbolic metamaterials
Source: Nat Commun. 2016 Feb 4;7:10568. doi: 10.1038/ncomms10568 (PMC4743008; doi:10.1038/ncomms10568)
Supplement: Supplementary Information — Supplementary Figures 1-6, Supplementary Notes 1-5 and Supplementary References. [file ncomms10568-s1.pdf]

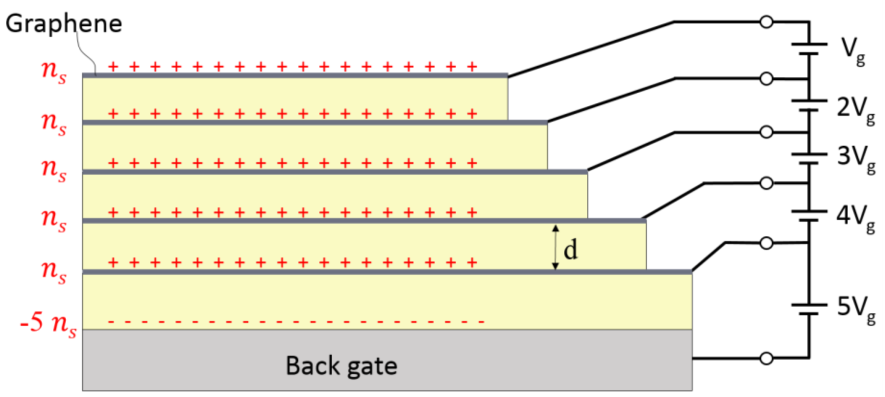

Supplementary Figure 1: **A potential scheme to electrically gate the graphene-based metamaterial.** Here n_s is the surface charge density. The voltage V_g equals $n_s d/\varepsilon$, where ε is the DC permittivity of the dielectric.

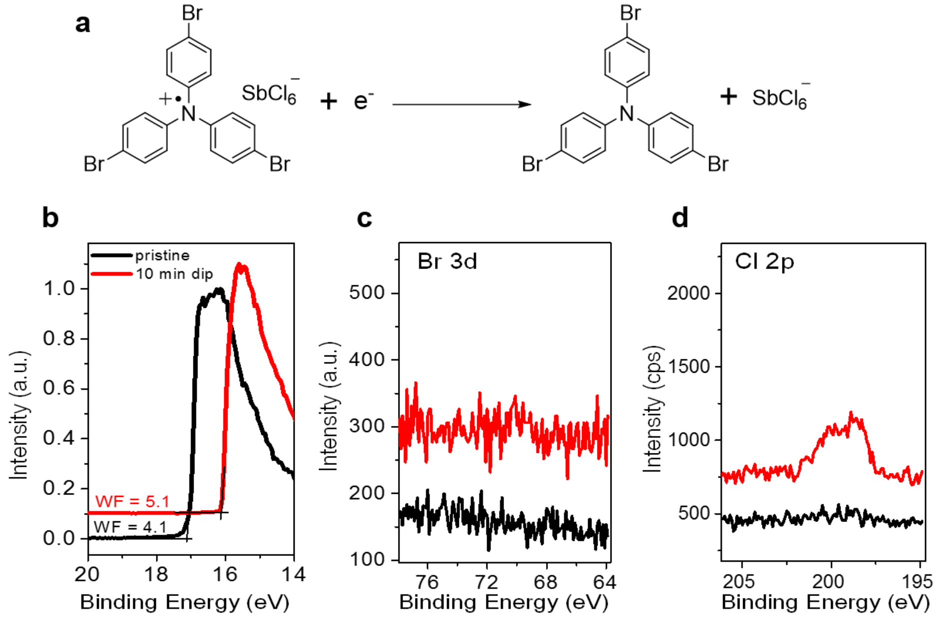

Supplementary Figure 2: **Characterization of the chemical doping.** (a) Doping reaction for "magic blue". (b) The secondary electron edge of graphene after 10 min treatment. (c-d) X-ray photoelectron spectroscopy core level spectra of Br 3d and Cl 2p. The black curves are the pristine graphene spectra, and the red curves are the spectra of graphene doped for 10 min in a 2.5 mM dopant solution.

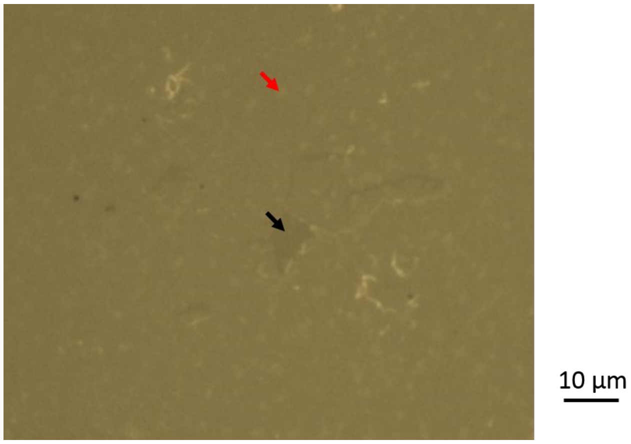

Supplementary Figure 3: **Optical image of monolayer CVD graphene transferred on a CaF_2 substrate.** The red and black arrows indicate the multilayer patch and the hole, respectively.

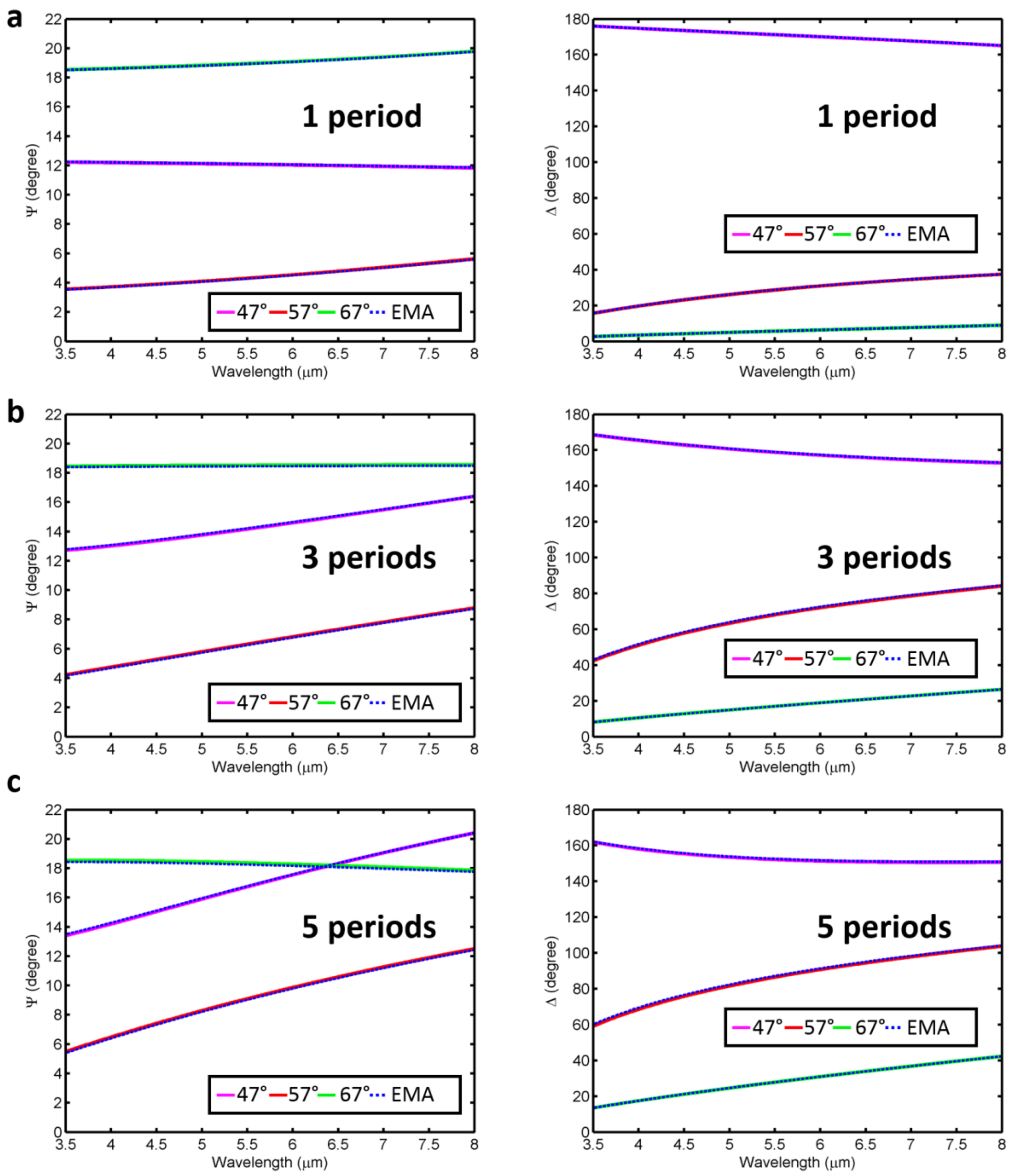

Supplementary Figure 4: **Calculation of ellipsometric angles Ψ and Δ for 1-period, 3-period and 5-period structures.** (a), (b), and (c) correspond to 1-period, 3-period and 5-period structures, respectively. The calculation is performed for incident angles of 47º, 57 º and 67 º. For all structures, the solid lines, which are calculated with exact transfer-matrix method, match very well with the dash lines, which are obtained under the EMA. The parameters used in the calculation are the same as Fig. 5 in the main text.

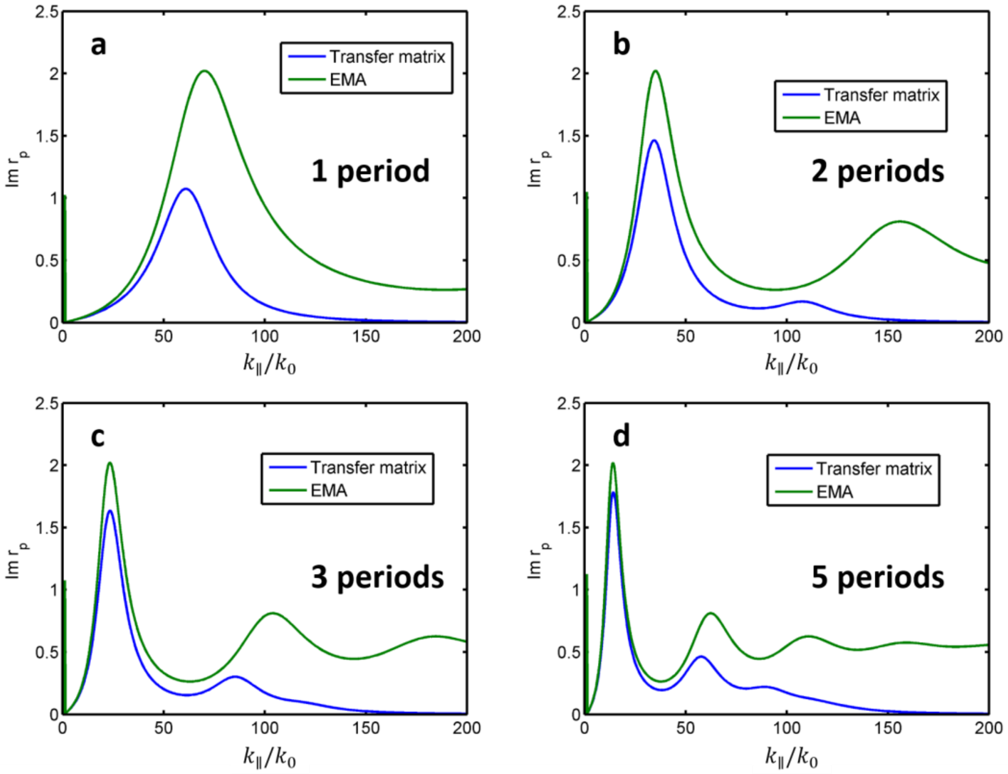

Supplementary Figure 5: **Calculation of Im r_p, the imaginary part of the Fresnel reflection coefficient, in the high-k regime.** (a), (b), (c) and (d) correspond to 1-period, 2-period, 3-period and 5-period structures, respectively. The blue and green lines are calculated by the transfer-matrix method and EMA respectively. The parameters used in the calculation are the same as Fig. 5 in the main text. The wavelength is 7 μm.

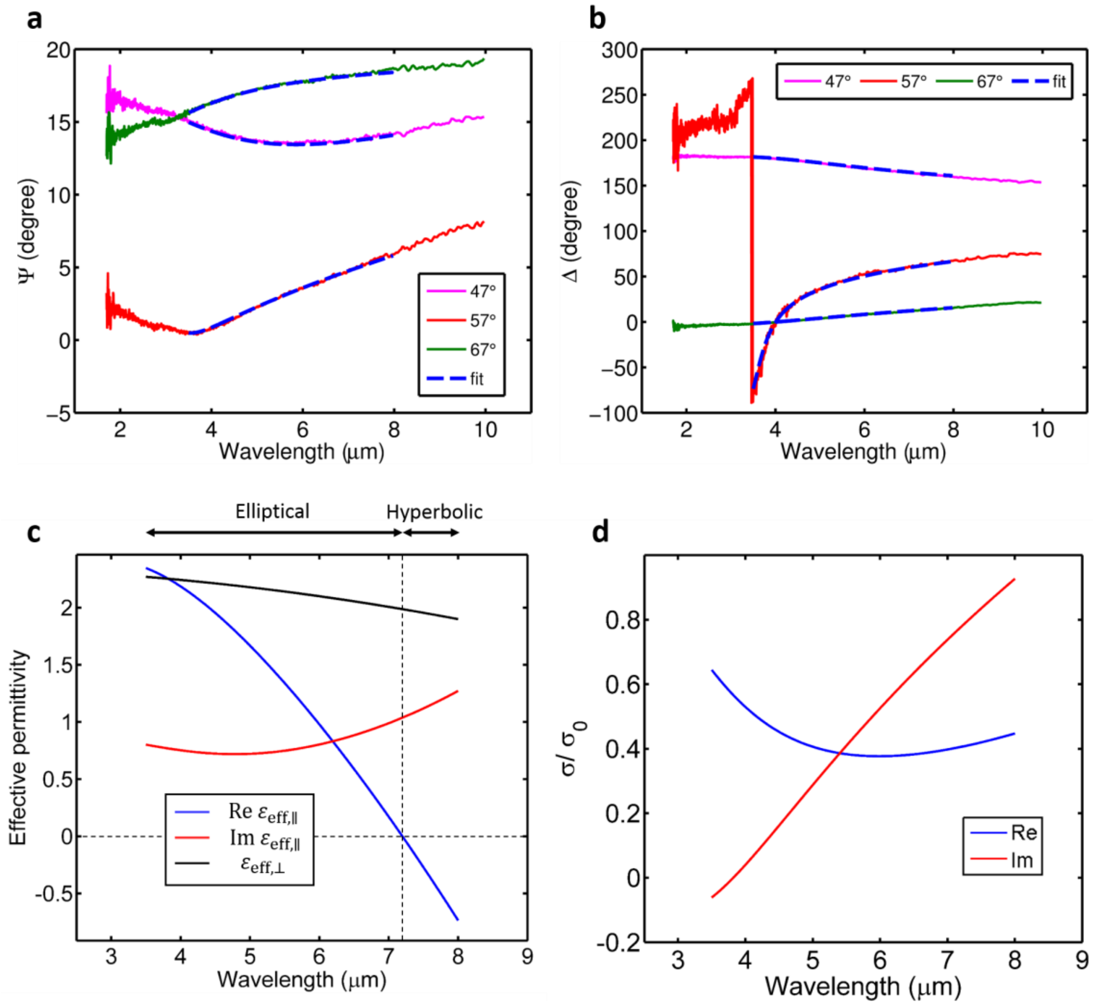

Supplementary Figure 6: **Extraction of the effective permittivity of the metamaterial incorporating unintentionally-doped CVD graphene. (**a) and (b) The ellipsometric angles Ψ and Δ acquired from the graphene-dielectric multilayer structure, respectively. The measurement is performed at incident angles of 47º, 57 º and 67 º. The blue dash lines show the fitting by homogenizing the multilayer structure into a metamaterial with the effective permittivities given by equation (1) in the main text. We extract from the fitting that E_{FC}=166 meV, s=47 meV and $\hbar\gamma$=44 meV. (c) The extracted effective permittivity of the metamaterial, which exhibits a topological transition from elliptical to hyperbolic dispersion at 7.2 µm. When the wavelength is at 8 µm, $\varepsilon_{\text{eff},\parallel}$ equals - 0.74+1.27i and $\varepsilon_{\text{eff},\perp}$ equals 1.9. (d) The extracted optical conductivity of the constituent CVD graphene in the metamaterial.

Supplementary Notes

Supplementary Note 1: **Electrical gating of graphene-based metamaterials.** Graphene offers a potentially unique building block for metamaterials especially because of the ability to control its conductivity electrically. Electrical gating is commonly used in graphene-based field-effect transistors (FETs), in which the carrier concentration of one graphene layer is controlled by the gate voltage. A similar idea can also apply to graphene-based metamaterials with multiple graphene layers. In Supplementary Fig. 1 we show how the electrical gating can potentially be applied in graphene-based metamaterials.

In order to gate the metamaterial such that the carrier concentration of all graphene layers change together, different voltages need to be applied to each graphene layer. Application of a single voltage to a top contact would result in an inhomogeneous density distribution due to interlayer screening [1]. Because the CVD graphene-based metamaterial studied in this work is fabricated by a layer-transfer method, each graphene layer could be accessed independently by patterning with photolithography. It can be calculated from electrostatics that the voltage profile shown in supplementary Fig. 1 can induce the same surface charge density n_s in all layers.

Supplementary Note 2: **Characterization of the chemical doping.** Supplementary Fig. 2a shows the doping reaction. Ultraviolet and x-ray photoelectron spectroscopy (UPS and XPS, respectively) are used to investigate the doping effects of "magic blue" on graphene. The work function ϕ is determined by UPS. Supplementary Fig. 2b shows the high binding energy cutoff regions of the UPS spectra for pristine and chemically-doped graphene. The secondary electron edge (SEE) shifts to lower binding energy upon dopant treatment. The work function is given by the difference between the energy of the UV photons (21.21 eV for the He I radiation used here) and the binding energy of the SEE. The work function increases significantly from 4.1 eV (pristine) to 5.1 eV (10 min treatment in a 2.5 mM dopant solution).

XPS is used to quantify the surface concentrations of "magic blue" (based on Cl 2p). During the doping process, tris(4-bromophenyl)ammoniumyl radical cation accepts an electron from the graphene to form neutral tris(4-bromophenyl)amine, which is assumed to remain in solution and/or to be washed away in the following rinsing step, while the hexachloroantimonate counter ion is left to balance the charges introduced to the film. This is supported by the fact that longer doping treatments lead to significant growth of the Cl 2p peak, but no obvious increase in the Br 3d peak, as shown in Supplementary Fig. 2c,d. Assuming all the dopants are fully reacted (only anions are present on the surface), the coverage can be calculated based on the dopant to graphene ratio relative to the theoretical value, which is estimated from how many dopant monomers can fit in a close packed arrangement on the surface of graphene. Thus, the concentration of "magic blue" can be deduced from the Cl/C ratio. The close-packed monolayer model for "magic blue" and calculation methods are described in our previous work [2,3]. The unit cell is viewed as a rhombus, and its area is calculated to be 60.2 $Å^2$. Since each ion contains six Cl atoms, by comparison with graphene, for which the unit cell with 2 carbons has an area of 5.24 $Å^2$, we can estimate that $\mathrm{Cl/C_{graphene}}$ = 0.26. The theoretical monolayer coverage of magic blue doping product on graphene can be compared with the XPS experimental data. We obtain a coverage of 62 ± 3 % of a molecular monolayer for a 10 min immersion in a 2.5 mM solution, which is indeed a sub-monolayer.

Supplementary Note 3: **Multilayer patches and holes in CVD graphene.** In practice, CVD graphene obtained experimentally is not a perfect and continuous monolayer. Supplementary Fig. 3 shows an image of CVD graphene transferred to a CaF_2 substrate taken in an optical microscope. Although most of the area is monolayer graphene, there are inevitably some multilayer patches (indicated by the red arrow) and some holes (indicated by the black arrow) [4]. Therefore, instead of being perfectly monolayer, a real sample of large-area CVD graphene is a combination of predominantly monolayer with some zero layer and multilayers.

Supplementary Note 4: **The effect of number of periods in graphene hyperbolic metamaterials.** In our experimental realization of graphene HMM, we have chosen to make samples with 5 periods of the graphene-dielectric unit cells. We have also shown in Fig. 4 of the main text that the 5-period structure can be accurately approximated as an effective medium described by the EMA formula. In fact, in the low-k regime, even one period of the graphene-dielectric unit cell can be homogenized by the same EMA formula and still reproduce the optical properties accurately. This can be shown by Supplementary Fig. 4, in which we calculate ellipsometric angles Ψ and Δ of 1-period, 3-period and 5-period samples using exact transfer-matrix method and the EMA formula Eq. (1) in the main text. The remarkable match between transfer-matrix and EMA results can be attributed to the fact that the period is very small compared to the mid-infrared wavelength ($d/\lambda <$ 1/300 in our case) such that the quasi-static limit is reached satisfactorily. This result demonstrates that the effective permittivities of graphene HMM retrieved from a low-k measurement such as ellipsometry are independent of the number of layers. Similar result showing that low-k optical properties are insensitive to the number of periods has also been reported in HMM consisting of metal-dielectric multilayers [5]. This is very different from what has been reported for fishnet negative-index metamaterials [6,7], in which the coupling between layers can in some cases significantly alters the effective parameters, and therefore a sufficient number of layers must be chosen in order to reach the convergence of effective optical properties.

In spite of the high accuracy of the EMA in the low-k regime for the graphene HMM, the high-k regime is where the real interest of HMM lies, as the enhanced photonic density of states and subwavelength imaging all rely on the existence of propagating high-k modes. Therefore, it is necessary to examine the EMA in the high-k regime [8], and in particular we can investigate how the number of periods influences the high-k optical properties. The existence of propagating high-k modes in HMM can be manifested by a nonzero Im r_p, the imaginary part of the Fresnel reflection coefficients for p-light [8,9], which is directly connected to the Purcell

factor. In Supplementary Fig. 5 we show the calculation of Im r_p in the high-k regime as the function of the in-plane k-vector for 1-period, 2-period, 3-period and 5-period structures. As shown by all panels in Supplementary Fig. 5, the EMA overestimates Im r_p, and the correct value calculated by the transfer-matrix method experiences a cut-off for large $k_\parallel/k_0$. This is because when $k_\parallel/k_0$ becomes large enough, the assumption that the period is much smaller than the wavelength eventually breaks down. Comparing different panels in Supplementary Fig. 5, we see that by increasing the number of layers, the results from the transfer-matrix method become closer to EMA calculations before the cut-off. Our choice of 5 periods can provide optical properties reasonably close to the effective medium up to $k_\parallel \approx 50k_0$. The calculation of Purcell factor in our previous work also shows that the Purcell factor reaches saturation with 5 periods [9].

Supplementary Note 5: **Hyperbolic metamaterial incorporating unintentionally-doped graphene.** For comparison, we have also fabricated another HMM sample with the same structure described in the main text, except that the CVD graphene in this sample is not chemically doped. The unintentionally CVD graphene is still lightly p-doped due to the adsorbed gas molecules and residual ammonium persulfate.

Supplementary Fig. 6a,b show the acquired ellipsometric angles Ψ and Δ of this sample. In fitting the data of this sample, we have found that we cannot obtain a satisfactory fit with the same procedure described in the main text. This is because the optical conductivity of lightly-doped graphene is not purely intraband even at mid-infrared wavelengths, and therefore Eq. (3) in the main text cannot describe well an actual graphene sample when there is an inhomogeneity in the Fermi energy. To account for the Fermi energy inhomogeneity, we assume the distribution in Fermi energy throughout the sample can be approximated by a Gaussian distribution, and fit the ellipsometric data with 3 free parameters: E_FC, s and γ. E_FC and s are the center and standard deviation of the Gaussian distribution that describes the inhomogeneity of Fermi energy. With these 3 free parameters, we can obtain a satisfactory fit, as shown in Supplementary Fig. 6a,b. The extracted values are E_FC=166 meV, s=47 meV and $\hbar\gamma$=44 meV. The extracted effective permittivities of this metamaterial and the optical conductivity of the constituent graphene are plotted in Supplementary Fig. 6c,d.

As expected, the optical topological transition wavelength of this metamaterial sample is red-shifted compared to the chemically-doped sample reported in the main text. This sample is hyperbolic for wavelengths longer than 7.2 µm. However, because of the intraband scattering, unintentionally-doped CVD graphene exhibits more material loss at this wavelength, and therefore the imaginary part of $\varepsilon_{\mathrm{eff},\parallel}$ is larger than its real part. In terms of the material loss of a HMM, the unintentionally-doped sample clearly is not as good as the chemically-doped sample reported in the main text.

Supplementary References

[1] Sun, D., Divin, C., Berger, C., De Heer, W. A., First, P. N. & Norris, T. B. Spectroscopic measurement of interlayer screening in multilayer epitaxial graphene. *Phys. Rev. Lett.* **104,** 136802 (2010).

[2] Tarasov, A. *et. al.* Controlled Doping of Large-Area Trilayer MoS_2 with Molecular Reductants and Oxidants. *Adv. Mater.* **27**, 1175 (2015).

[3] Paniagua, S. A. *et. al.* Production of heavily n-and p-doped CVD graphene with solution-processed redox-active metal–organic species. *Mater. Horiz.* **1**, 111 (2014).

[4] Liang, X. *et. al.* Toward clean and crackless transfer of graphene. *ACS nano* **5,** 9144-9153 (2011).

[5] Cortes, C. L., Newman, W., Molesky, S. & Jacob, Z. Quantum nanophotonics using hyperbolic metamaterials. *Journal of Optics* **14,** 063001 (2012).

[6] Soukoulis, C. M. & Wegener, M. Past achievements and future challenges in the development of three-dimensional photonic metamaterials. *Nature Photonics* **5,** 523-530 (2011).

[7] Zhou, J., Koschny, T., Kafesaki, M. & Soukoulis, C. M. Negative refractive index response of weakly and strongly coupled optical metamaterials. *Phys. Rev. B* **80,** 035109 (2009).

[8] Kidwai, O., Zhukovsky, S. V. & Sipe, J. E. Effective-medium approach to planar multilayer hyperbolic metamaterials: Strengths and limitations. *Phys. Rev. A* **85,** 053842 (2012).

[9] DaSilva, A. M., Chang, Y. C., Norris, T. B. & MacDonald, A. H. Enhancement of photonic density of states in finite graphene multilayers. *Phys. Rev. B* **88,** 195411 (2013).
